# Maternal and child health interventions in Nigeria: a systematic review of published studies from 1990 to date

**DOI:** 10.1186/1472-6963-14-S2-P4

**Published:** 2014-07-07

**Authors:** Musa Abubakar Kana, Henry Doctor, Bàrbara Peleteiro, Nuno Lunet, Henrique Barros

**Affiliations:** 1 Department of Community Medicine, Faculty of Medicine, Kaduna State University, Kaduna, Kaduna State, Nigeria; 2 Institute of Public Health, University of Porto (ISPUP), Porto, Portugal; 3 United Nations Office on Drugs and Crime, Abuja, Nigeria; 4 Department of Clinical Epidemiology, Predictive Medicine and Public Health, University of Porto Medical School, Porto, Portugal

## Background

Poor maternal and child health indicators have been reported in Nigeria since the 1990s. Many health system interventions have been instituted to reverse the trend and ensure that Nigeria is on track to achieve the Millennium Development Goals. This systematic review aims to describe the Maternal, Newborn and Child Health (MNCH) interventions that have been implemented in Nigeria since the 1990s.

## Methods

PubMed was searched from inception in 1990 up to January 2014 to identify reports of interventions targeting MNCH in Nigeria.

## Results

We identified 33 eligible studies from 835 studies, after applying pre-defined exclusion criteria to title/abstract and full text. Two interventions included all regions of the country whereas the others were conducted at the regional level, most of them in a rural setting. All included studies presented a quasi-experimental design and most of them using a community-based (n=24) or a hospital-based (n=8) approach. Most of the interventions targeted women of childbearing age, under-five children, and health system issues. The outcomes of the different interventions included maternal health promotion (family planning, antenatal care, and prevention of mother-to-child transmission (PMTCT)), prevention of obstetric complications (safe management of ante and postpartum, and clinical audit of quality of obstetrics services), child health promotion (immunization, and infant feeding), prevention of childhood diseases (home management of malaria, insecticide treated nets (ITN)), and health system strengthening (policy for free MNCH services, electronic health information management system).

## Discussion/Conclusions

Heterogeneous approaches for the same target groups have been conducted. Most of the interventions lacked a control group thereby compromising the measurement of their effectiveness. The true impact of these strategies cannot be assessed unless by indirect methods, precluding the generalizability of the results. We hope that the results of this systematic review will provide more insights on areas of improvement in order to achieve better health for all Nigerians particularly for women and children.

